# Phylogenetic relationships of freshwater fishes of the genus *Capoeta* (Actinopterygii, Cyprinidae) in Iran

**DOI:** 10.1002/ece3.2411

**Published:** 2016-10-20

**Authors:** Hamid Reza Ghanavi, Elena G. Gonzalez, Ignacio Doadrio

**Affiliations:** ^1^ Museo Nacional de Ciencias NaturalesBiodiversity and Evolutionary Biology DepartmentCSICMadridSpain

**Keywords:** biodiversity, cyprinid, freshwater fish species, Iran, Middle East, phylogeography

## Abstract

The Middle East contains a great diversity of *Capoeta* species, but their taxonomy remains poorly described. We used mitochondrial history to examine diversity of the algae‐scraping cyprinid *Capoeta* in Iran, applying the species‐delimiting approaches General Mixed Yule‐Coalescent (GMYC) and Poisson Tree Process (PTP) as well as haplotype network analyses. Using the BEAST program, we also examined temporal divergence patterns of *Capoeta*. The monophyly of the genus and the existence of three previously described main clades (Mesopotamian, Anatolian‐Iranian, and Aralo‐Caspian) were confirmed. However, the phylogeny proposed novel taxonomic findings within *Capoeta*. Results of GMYC, bPTP, and phylogenetic analyses were similar and suggested that species diversity in Iran is currently underestimated. At least four candidate species, *Capoeta *sp4, *Capoeta *sp5, *Capoeta* sp6, and *Capoeta *sp7, are awaiting description. *Capoeta capoeta* comprises a species complex with distinct genetic lineages. The divergence times of the three main *Capoeta* clades are estimated to have occurred around 15.6–12.4 Mya, consistent with a Mio‐Pleistocene origin of the diversity of *Capoeta* in Iran. The changes in Caspian Sea levels associated with climate fluctuations and geomorphological events such as the uplift of the Zagros and Alborz Mountains may account for the complex speciation patterns in *Capoeta* in Iran.

## 1 | INTRODUCTION

The genus *Capoeta* Valenciennes in Cuvier & Valenciennes 1842, comprising more than 20 species (Froese & Pauly, 2016), is a major component of Iranian freshwater fauna and is widespread throughout western Asia from Anatolia to the Levant, Transcaucasia, the Tigris and Euphrates basins, Turkmenistan, and northern Afghanistan (Bănărescu, 1999; Levin et al., 2012). Several studies examining the taxonomy and relationships of *Capoeta* have found it to be monophyletic and most closely related to the Euro-Mediterranean barbels of the genus *Luciobarbus* Heckel 1843 (Levin et al., 2012; Machordom & Doadrio, 2001b). *Capoeta* species are hexaploid, and their origin has been postulated to be in an ancient hybridization event between tetraploid *Luciobarbus* and diploid *Cyprinion* (Yang et al., 2015). Genetic variation within *Capoeta* has not been studied in detail, and evidence indicates that the taxonomic status of some groups needs confirmation (Bektaş, Çiftçi, Eroğlu, & Beldüz, 2011; Levin et al., 2012; Tsigenopoulos, Durand, Ünlü, & Berrebi, 2003; Turan, 2008).

Iran presents a complex biogeography within the Palearctic region, enriched by the influence of Indo-Malayan and African ichthyofauna (Armantrout, 1980; Coad, 2006, 2015; Coad & Vilenkin, 2004). The origin and dispersion of freshwater fauna of Iran is a matter of debate (Coad, 2006; Coad & Vilenkin, 2004; Durand, Tsigenopoulos, Unlü, & Berrebi, 2002; Heller, 2007; Perea et al., 2010). One hypothesis suggests that the freshwater fauna with low salinity affinity dispersed from the Middle East northward to the Paratethys and then westward into Europe and eastward into western Asia (Durand et al., 2002; Heller, 2007; Por & Dimentman, 1985, 1989). A second hypothesis proposes that, prior to Pliocene orogenesis, the proto-Euphrates collected freshwater from the Middle East and maintained contact with the Black and Caspian Seas (Durand et al., 2002). More recent studies suggest that the colonization of Europe by Leuciscinae most likely occurred from southwestern Asia via the Balkanian/Anatolian/Iranian landmass in the Early Oligocene (Perea et al., 2010).

There is no consensus on the status of freshwater biodiversity of the Iranian region. While some authors suggest low-to-medium freshwater biodiversity and endemism (Abell et al., 2008), others consider the values to be high (Coad, 2006). The diversity of its freshwater ichthyofauna has not been well studied, and most of the communities and their associated diversity are poorly characterized. Recent publication of numerous taxonomic studies describing new species in Iran indicates the importance of undertaking further study of its ichthyofauna (Coad & Bogutskaya, 2009; Esmaeili, Sayyadzadeh, Özulug, Geiger, & Freyhof, 2014; Esmaeili, Teimori, Gholami, & Reichenbacher, 2014; Freyhof, Esmaeili, Sayyadzadeh, & Geiger, 2014; Golzarianpour, Abdoli, & Freyhof, 2011; Golzarianpour, Abdoli, Patimar, & Freyhof, 2013; Mousavi-Sabet, Vasil'eva, Vatandoust, & Vasil'ev, 2011; Mousavi-Sabet, Vatandoust, & Doadrio, 2015; Teimori, Esmaeili, Erpenbeck, & Reichenbacher, 2014; Teimori, Schulz-Mirbach, Esmaeili, & Reichenbacher, 2012; Zareian, Esmaeili, & Freyhof, 2016).

The genus *Capoeta* may be an ideal model for study of the biogeographical and evolutionary history of the freshwater fauna of Iran, given its countrywide distribution and the extensive variation in habitats occupied, from high-mountain crystalline streams to deep lowland/coastal muddy rivers (Bănărescu, 1999). Being mostly algae-scrappers, these species depend mostly on clear and not very deep rivers where light is not a limitation to the growth of algae. Some species are placed as critically endangered (*C. pestai* and *C. angorae*), many as endangered (*C. antalyensis*, *C. barroisi*, *C. bergamae*, *C. damascina*, *C. kosswigi*, *C. sieboldi* and *C. tinca*) but also many as data deficient in Turkey by Fricke, Bilecenoğlu, and Sarı (2007). In Iran, *C. capoeta* is considered of least concern in Caspian basin by Kiabi, Abdoli, and Naderi (1999), but in general, there are not strong assessments on their conservation status and more studies are needed. Main threats for this species seem to be habitat loss, water abstraction, construction measures, and pollution and probably on a lower degree invasive species. On the other hand, many species were considered very widely distributed which recent taxonomic studies limit their distribution and describe new more locally limited species which suggest a higher conservation status for them and show the urgent need of studies on the conservation status of generally all freshwater fishes in Iran.

Members identified as belonging to this genus are present in all Iranian basins (probably with the exception of southeastern ones), so their global distribution comprises a wide region from Syria, Lebanon, and Turkey in the west to Turkmenistan, Afghanistan, and probably northern Pakistan in the east, Georgia and southwestern Russia in the North, and Iranian shores of Persian Gulf in the south (Bănărescu, 1999; Coad, 2015). Recent studies of the genus are mainly taxonomic, including the description of new species (Esmaeili, Zareian, Eagderi, & Alwan, 2016; Özulug & Freyhof, 2008; Turan, Kottelat, & Ekmekçi, 2008; Zareian, Esmaeili, & Freyhof, 2016), or are ecological, such as evaluations of their value as biomarkers to assess human impact on aquatic environments (Anvarifar et al., 2011, 2013; Ebrahimi & Taherianfard, 2010; Fallah, Nematollahi, & Saei-Dehkordi, 2013; Faradonbe, Eagderi, & Moradi, 2015; Johari, Coad, Mazloomi, Kheyri, & Asghari, 2009; Patimar & Mohammadzadeh, 2011; Samaee, Patzner, & Mansour, 2009). Little is known of the genetic variation in extant members of the genus or the diversification patterns that shaped its current diversity.

This study investigated the phylogeny of the main freshwater populations of *Capoeta*, including the most complete dataset available, and provided a hypothesis on the evolutionary history and diversification of the genus in the region. The primary aims of this study were (1) to assess species boundaries within *Capoeta* and evaluate cryptic diversity and species endemism by sequencing the cytochrome *b* gene; (2) to investigate the phylogeny within *Capoeta* species based on geographic sampling; and (3) to propose a hypothesis for the origin of freshwater fauna of Iran considering possible vicariant events that may have shaped the diversity of the genus.

## 2 | MATERIAL AND METHODS

### 2.1 | Sample collection

Three hundred and five specimens of the genus *Capoeta* and one specimen of *Barbus lacerta* were collected by electrofishing at 47 sites in 13 river basins, covering most of its distribution in the country (Table 1; Fig. 1) with local authority permission. A fragment of pelvic fin was cut and stored in microtubes in 96% ethanol and deposited in the Tissue and DNA Collection of the National Museum of Natural Sciences of Madrid (MNCN-CSIC), Spain. Few fish from each site were killed with overdoses of MS222, fixed in 8% formalin, and later preserved in 70% ethanol in the Ichthyology collection of MNCN-CSIC, Spain.

### 2.2 | DNA extraction, amplification, and sequencing

DNA was extracted using the DNeasy® Blood & Tissue Kit (QIAGEN, Hilden, Germany). The entire cytochrome *b* (*cytb*) gene (1140 bp) was amplified by polymerase chain reaction (PCR) according to the protocol described in Perdices and Doadrio (2001) using GluDG.L and H16460 primers. Briefly, DNA was amplified in 25 µl reactions [1× buffer, 1.5 µM MgCl2, 0.5 mM of each primer, 0.2 µM dNTP of each nucleotide, 17.55 µl dd$H_2O$, 1 µl template DNA, and 1U Taq polymerase (Biotools, Madrid, Spain)]. PCR was performed at 95°C (2 min) followed by 30 cycles of 95°C for 30 s, 54°C or 1 min 20 s, 72°C for 2 min 20 s, and a final extension of 10 min at 72°C. PCR products were visualized on 1.5% agarose gels and later purified by ethanol. Both strands were sequenced using the service of Macrogen Inc. (Seoul, Korea).

Alignments of nucleotide sequences were constructed with CLUSTAL W using default parameters (Larkin et al., 2007), or with Geneious software (Geneious v. 8.0.3; Biomatters, http://www.geneious.com/),

**TABLE 1** Sampling sites and coordinates. Numbers in the first column (Loc) correspond to numbers on the sampling map in Fig. 1

| | | | | | | Clade A: Mesopotamian | | | Clade B: Aralo-Caspian | | | | | | Clade C: Anatolian-Iranian | | | | | | | |
|---|---|---|---|---|---|---|---|---|---|---|---|---|---|---|---|---|---|---|---|---|---|---|
| Loc | River | Basin | Locality | GPS coordinates | Alt. (m) | *C. mandica* | *C. trutta* | *C. aculeata* | *C. aff aculeata* | *C. capoeta* | *C. heratensis* | *C. sp1* | *C. sp6* | *C. sp7* | *C. coadi* | *C. buhsei* | *C. damascina* | *C. saadii* | *C. sp3* | *C. sp4* | *C. sp5* | Total number of species per basin |
| 1 | SEFID RUD | CASPIAN | LOWSHAN | N36.637033 E49.48825 | 321 | | | | | | | 7 | | | | | | | | | | 3 |
| 2 | TEJAN | | PAYIN HULAR (SARI) | N36.486733 E53.08625 | 92 | | | | | | | 3 | | 4 | | | | | | | | |
| 3 | GHEZEL OZAN | | NESAREH | N35.870208 E47.081785 | 1739 | | | | | | | 18 | | | | | | | | | | |
| 4 | NA | | CHOOBAR | N38.176768 E48.881804 | 23 | | | | | | | 10 | | | | | | | | | | |
| 5 | NA | | NA | N38.030870 E48.892113 | 2 | | | | | | | 8 | | | | | | | | | | |
| 6 | GORGAN RUD | | TALESH | N37.800597 E48.884633 | 70 | | | | | 1 | | 10 | | | | | | | | | | |
| 7 | NA | | ASALEM | N37.714893 E48.928986 | 92 | | | | | | | 6 | | | | | | | | | | |
| 8 | SHAFA RUD | | PUNEL | N37.531170 E49.110149 | 50 | | | | | | | 8 | | | | | | | | | | |
| 9 | CHALK RUD | | KATALOM | N36.872128 E50.771551 | −16 | | | | | | | 6 | | | | | | | | | | |
| 10 | NA | | KELARABAD | N36.701598 E51.219531 | 30 | | | | | | | 6 | | | | | | | | | | |
| 11 | ANGUETA RUD | | SANGETAB | N36.477021 E52.225348 | 38 | | | | | | | 5 | | | | | | | | | | |
| 12 | ATRAK | | MARAVEH TAPPEH | N37.908511 E55.952827 | 208 | | | | | | | 6 | | | | | | | | | | |
| 13 | GOLESTAN | | TANGRAH | N37.382139 E55.853458 | 538 | | | | | | | 6 | | | | | | | | | | |
| 14 | SHAPUR | DALAKI | BISHAPUR | N29.781733 E51.57945 | 820 | | | | | | | | | | | | | 3 | | | | 1 |
| 15 | SHAPUR | | BISHAPUR | N29.781733 E51.57945 | 820 | | | | | | | | | | | | | 7 | | | | |
| 16 | KHORRAM RUD | KARKHEH (TIGRIS) | KHORRAMABAD | N33.597517 E48.297267 | 1287 | | | | 3 | | | | | | | | | | 3 | | | 2 |
| 17 | BAD AVAR | | NURABAD | N34.080783 E48.0027 | 1798 | | | | | | | | | | | | | | 1 | | | |
| 18 | SAR POL | | KANGAVAR | N34.46815 E47.9341 | 1462 | | | | 2 | | | | | | | | | | 5 | | | |
| 19 | KHORRAM RUD | | ARAN | N34.414914 E47.920146 | 1429 | | | | 4 | | | | | | | | | | | | | |
| 20 | GAMASIAB | | BISOTUN | N34.388516 E47.437708 | 1286 | | | | | | | | | | | | | | 2 | | | |
| 21 | NA | | KAMYARAN | N34.713421 E46.889624 | 1336 | | | | | | | | | | | | | | 7 | | | |
| 22 | SIRWAN | SIRWAN (TIGRIS) | GAWANEH | N34.989180 E46.969513 | 1427 | | | | | | | | | | | | 3 | | | | | 1 |
| 23 | BESHAR | KARUN (TIGRIS) | YASUJ | N30.87285 E51.33435 | 1600 | | | 2 | | | | | | | 3 | | | | | | | 3 |
| 24 | DASHT-E RUM | | DASHT-E RUM (YASUJ) | N30.52425 E51.52395 | 2120 | | | | | | | | | | 7 | | | | | | | |
| 25 | BESHAR | | MADOVAN (YASUJ) | N30.835467 E51.354083 | 1582 | | | 6 | | | | | | | 5 | | | | | | | |
| 26 | KARUN | | LORDEGAN | N31.669769 E50.769861 | 1060 | | 3 | | | | | | | | 1 | | | | | | | |
| 27 | SARKHUN | | DEHNO | N31.711333 E50.589983 | 1449 | | | | | | | | | | 4 | | | | | | | |
| 28 | TIRE | DEZ (TIGRIS) | KAGHEH | N33.634417 E48.982117 | 1520 | | | | | | | | | | | | | | | 2 | | 1 |

(continues)

**TABLE 1** (continued)

| Loc | River | Basin | Locality | GPS coordinates | Alt. (m) | Clade A: Mesopotamian | | | Clade B: Aralo-Caspian | | | | | | Clade C: Anatolian-Iranian | | | | | | | Total number of species per basin |
|---|---|---|---|---|---|---|---|---|---|---|---|---|---|---|---|---|---|---|---|---|---|---|
| | | | | | | *C. mandica* | *C. trutta* | *C. aculeata* | *C. aff aculeata* | *C. capoeta* | *C. heratensis* | *C. sp1* | *C. sp6* | *C. sp7* | *C. coadi* | *C. buhsei* | *C. damascina* | *C. saadii* | *C. sp3* | *C. sp4* | *C. sp5* | |
| 29 | SHADKAM | KOR | QADER ABAD (FARS) | N30.4041 E53.234133 | 2086 | | | 3 | | | | | | | | | | 5 | | | | 2 |
| 30 | KERGHEH | MAND | EMAMZADEH DAVOD | N28.901317 E52.378967 | 1544 | | | | | | | | | | | | | 8 | | | | 2 |
| 31 | SHUR | | FIRUZABAD | N28.853233 E52.517167 | 1314 | 2 | | | | | | | | | | | | 5 | | | | |
| 32 | GHAREH AGHAJ | | KAVAR (FARS) | N29.181417 E52.692583 | 1516 | 6 | | | | | | | | | | | | 1 | | | | |
| 33 | ZAKHEH | | KHANEH ZENIAN | N29.678967 E52.14715 | 1970 | 3 | | | | | | | | | | | | 6 | | | | |
| 34 | NAMRUD | NAMAK | FIRUZKUH | N35.767083 E52.881233 | 2066 | | | | | | | | | | | 5 | | | | | | 2 |
| 35 | NAMRUD | | ARJOMAND | N35.767833 E52.55385 | 2007 | | | | | | | | 3 | | | 8 | | | | | | |
| 36 | NAMRUD | | FIRUZKUH | N35.761583 E52.8838 | 2090 | | | | | | | | | | | 7 | | | | | | |
| 37 | JAJRUD | | JAJRUD | N35.764644 E51.693300 | 1480 | | | | | | | | 7 | | | 6 | | | | | | |
| 38 | SOLEGHAN | | SOLEGHAN | N35.798809 E51.254693 | 1391 | | | | | | | | | | | 5 | | | | | | |
| 39 | NA | | TOUREH | N34.045640 E49.353837 | 1847 | | | | | | | | | | | 2 | | | | | | |
| 40 | ZAYANDEH RUD | ZAYANDEH RUD | HOJAT ABAD | N32.718367 E50.782967 | 2337 | | | | | | | | | | 2 | | | | | | | 1 |
| 41 | HARERIZ | ZOHREH | KHUMEH ZAR | N30.028 E51.560717 | 1026 | | | | | | | | | | | | | | | | 6 | 2 |
| 42 | SHUR / FAHLIAN | | FAHLIAN | N30.183683 E51.523617 | 940 | | 9 | | | | | | | | | | | | | | | |
| 43 | GADAR | ORUMIEH | OSHNAVIEH | N37.000522 E45.093113 | 1435 | | | | | 6 | | | | | | | | | | | | 1 |
| 44 | BARAN DUZ | | HASHEM ABAD | N37.276943 E44.898200 | 1480 | | | | | 6 | | | | | | | | | | | | |
| 45 | NAZLU CHAI | | SEROW | N37.699333 E44.742177 | 1513 | | | | | 6 | | | | | | | | | | | | |
| 46 | NA | TEDZHEN | ABGARM | N36.565865 E60.047762 | 939 | | | | | | 6 | | | | | | | | | | | 1 |
| 47 | NA | | GHALEH NOW | N36.794025 E59.950682 | 905 | | | | | | 5 | | | | | | | | | | | |
| Total number of analyzed samples per species | | | | | | 11 | 12 | 11 | 9 | 19 | 11 | 99 | 10 | 4 | 22 | 33 | 3 | 35 | 18 | 2 | 6 | |

NA = no known name for location or river. Numbers in species column = number of specimens.

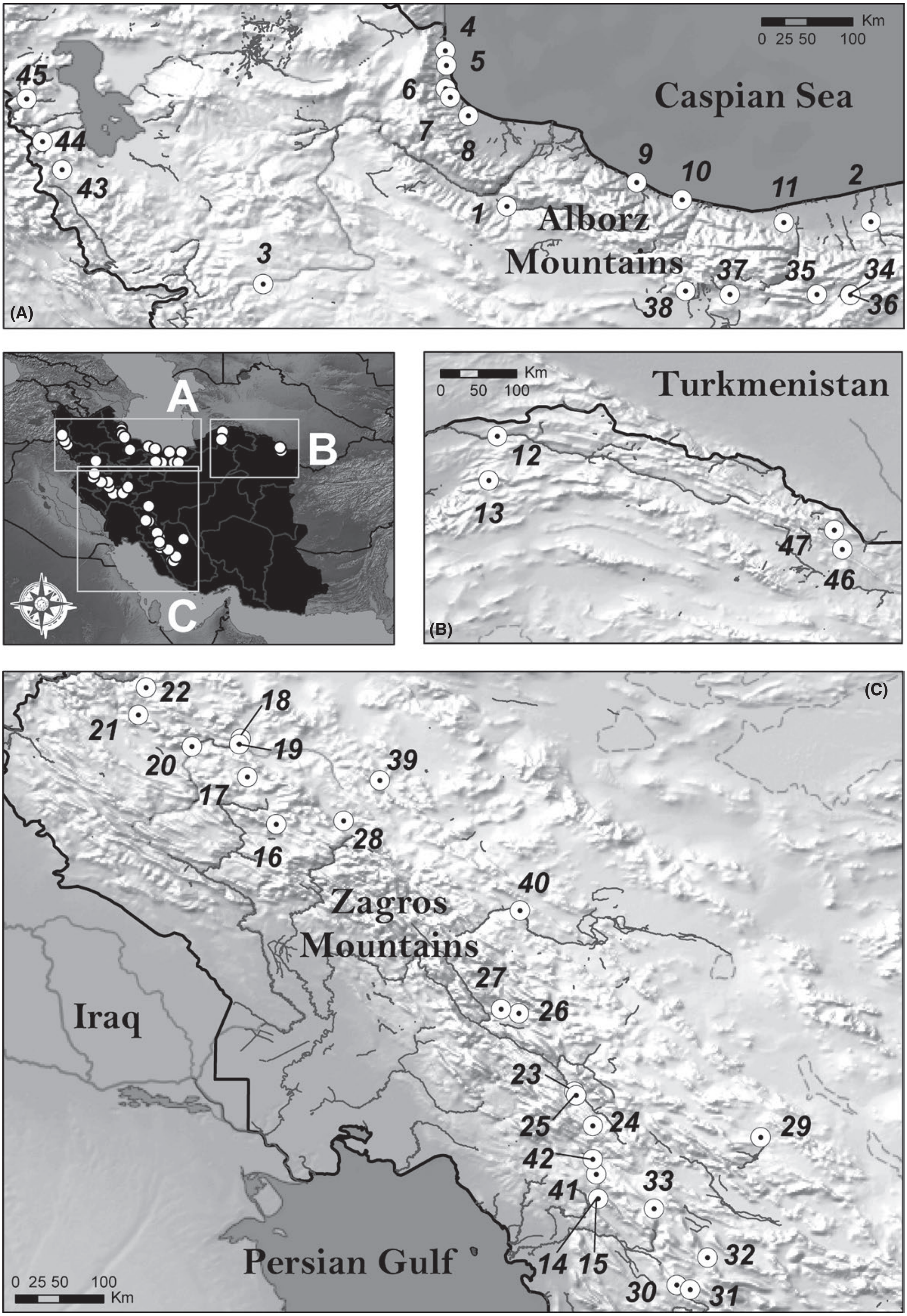

**FIGURE 1** Map of Iran and sampling points. Numbers of the sampling sites on the Figure correspond to the numbers of sampling sites on Table 1

and visually verified to maximize positional homology. Codification of amino acids was used to confirm the alignment and to avoid the inclusion of stop codons. Sequences of the complete *cytb* gene were trimmed to the size of the smallest fragment, and alignments produced a dataset of 1040 base pairs (bp).

The dataset used for phylogenetic inference included 439 *cytb* sequences, 306 of which we amplified and other 133 correspond to the homologous region available for *Capoeta* in GenBank. Sequences of *Luciobarbus subquincunciatus* (Günther 1868), *Luciobarbus brachycephalus* (Kessler 1872), and *Barbus lacerta* Heckel 1843, obtained from GenBank, were used as outgroup (Berrebi & Tsigenopoulos, 2003; Levin et al., 2012; Machordom & Doadrio, 2001a). All sequences and GenBank accession numbers are listed in Tables S1–S3.

## 2.3 | Data analysis

The sequences were collapsed to haplotypes using the program Alter (Glez-Peña, Gómez-Blanco, Reboiro-Jato, Fdez-Riverola, & Posada, 2010). Uncorrected-*p* pairwise distances between and within species (Table S4) were calculated with Mega 6 (Tamura, Stecher, Peterson, Filipski, & Kumar, 2013). A bootstrapping process was implemented with 1000 repetitions. As multiple tests, *p*-values were further adjusted by Bonferroni's correction (Rice, 1989).

The Akaike information criterion implemented in PartitionFinder v. 1.1.1 (Lanfear, Calcott, Ho, & Guindon, 2012) selected K80+I+G, F81+I, and GTR+I+G evolutionary models, considering each codon position as an independent partition. jModelTest 2.1.4 (Darriba, Taboada, Doallo, & Posada, 2012) selected GTR+I+G as the best evolutionary model for nonpartitioned sequence alignment. RAxML (Stamatakis, 2006) implemented in raxmlGUI 1.3 (Silvestro & Michalak, 2012) was used to estimate the maximum-likelihood (ML) tree using the selected evolutionary model for the sequence alignment. Bayesian inference was conducted with MrBAYES v. 3.2.2 (Ronquist et al., 2012). Two simultaneous analyses were run on $10^7$ generations, each with four MCMC chains sampling every 100 generations. Convergence was checked on Tracer 1.6 (Rambaut & Drummond, 2013). After discarding the first 10% of generations as burn-in, we obtained the 50% majority rule consensus tree and the posterior probabilities.

We reconstructed haplotype network sequences to resolve relationships among closely related haplotypes (Crandall, 1994).

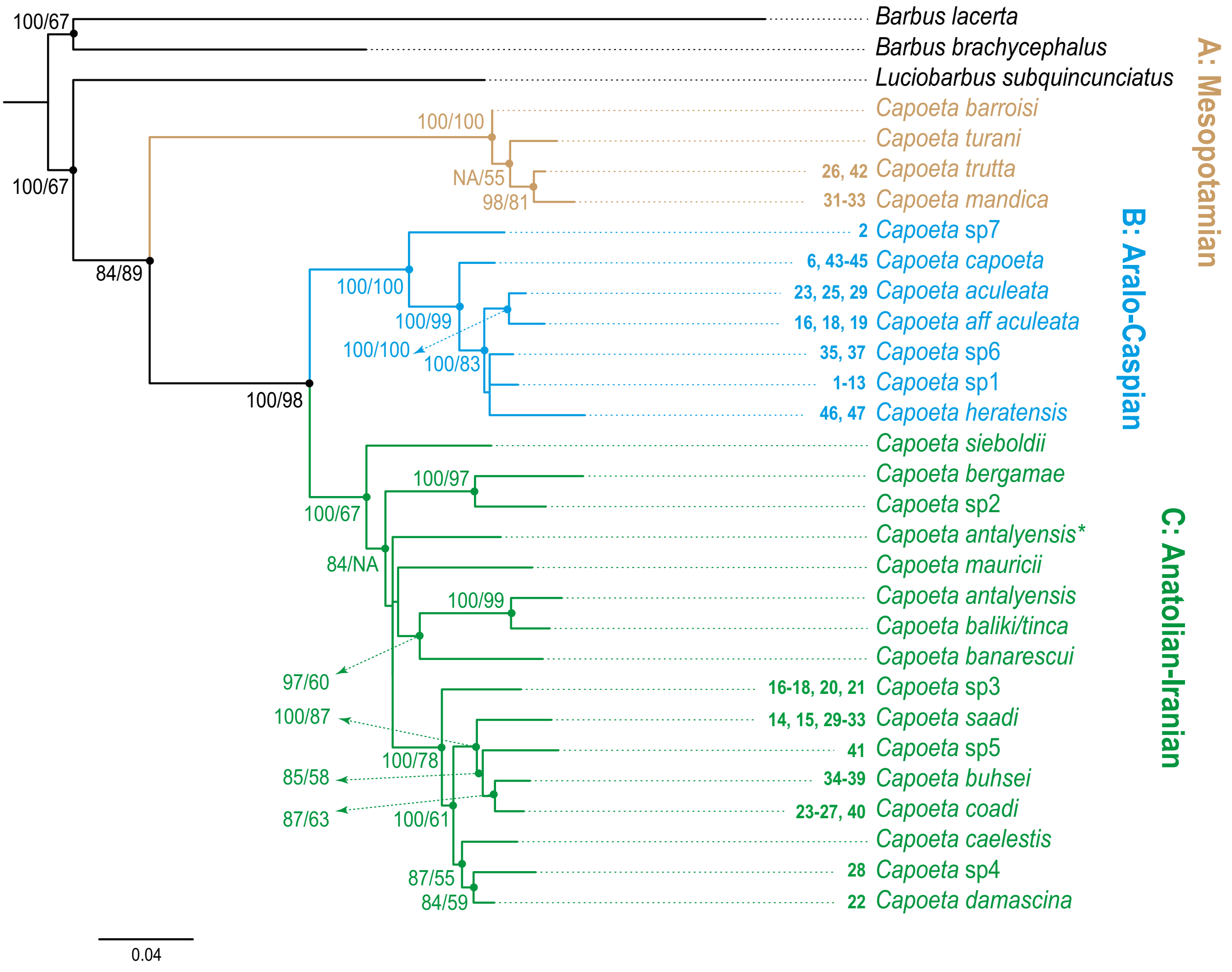

**FIGURE 2** *Capoeta* genus; three clades. Values at nodes correspond to BI posterior probability/ML bootstrap. Numbers before each species name corresponds to the locality code on Table 1 and Figure 1.

Haplotype genealogies in these clades were obtained by HaploView v. 4.2 (Barrett, Fry, Maller, & Daly, 2005).

To delimit species, two approaches were used. The Generalized Mixed Yule-Coalescent (GMYC) model, which uses a time-calibrated tree and delimits species on a divergence time basis (Fujisawa & Barraclough, 2013), and a Poisson tree process (PTP) model, using a distance-based tree to delimit species, implemented in Bayesian PTP (bPTP) (Zhang, Kapli, Pavlidis, & Stamatakis, 2013). Both GMYC and bPTP were accessed at Exelixis Labs (http://sco.h-its.org/exelixis/web/software/PTP/index.html).

Divergence times within *Capoeta* were estimated using a Bayesian relaxed molecular clock approach, implemented in BEAST v. 1.8 (Drummond, Suchard, Xie, & Rambaut, 2012). Dating analyses were performed using the evolutionary models described above. To calibrate the tree, an expanded sequence matrix was considered that included sequences of *Barbus* and *Luciobarbus* species (Table S3). For the molecular clock, multiple calibration points were based on fossil evidence of *Barbus* (18–19 Mya) and *Luciobarbus* genus (16.9–17.7 Mya) (Böhme & Ilg, 2003). The third calibration point considered was the Iberian Peninsula *Luciobarbus* species dated at 5.33–7.05 Mya (Doadrio & Casado, 1989; García-Alix, Minwer-Barakat, Martín Suárez, Freudenthal, & Martín, 2008). The branch rates were derived following an uncorrelated lognormal distribution and a Yule speciation prior (Drummond, Ho, Phillips, & Rambaut, 2006). Each final MCMC chain was run for $10^8$ generations (10% burn-in), with parameters sampled every 1000 steps. Output from BEAST was examined in Tracer 1.6 (Rambaut & Drummond, 2013), and the tree results were summarized using TreeAnnotator 1.7 (Drummond et al., 2012).

## 3 | RESULTS

Of the 1040 bp of partial mtDNA *cytb*, 744 were constant and 254 were parsimony informative. The Bayesian and ML analyses yielded essentially the same topologies with similar support (Fig. 2). The reconstructed topology was also in agreement with previously published higher level phylogenies that included *Capoeta* (Berrebi & Tsigenopoulos, 2003; Levin et al., 2012; Turan, 2008). The phylogeny results supported the monophyly of *Capoeta* and identified it as sister to *Luciobarbus*. Results revealed the presence of three well-supported clades: A—Mesopotamian; B-Aralo-Caspian; and C—Anatolian-Iranian, as has been proposed by other authors (Levin et al., 2012) (Fig. 2). Uncorrected-*p* genetic distances for the *cytb* gene were 8.7% for the pairwise comparison of clades A and B, 8.2% for clades A and C, and 6.2% between clades B and C. Genetic distances between and within species are listed in Table S4.

### 3.1 | Mesopotamian clade

The Mesopotamian clade was the basal clade within *Capoeta* with high support values, primarily comprising species from the Tigris and Euphrates Rivers together with several river basins in southwestern Iran (Figs 2 and 3). Results suggested three well-supported subclades that corresponded to *Capoeta trutta* (Heckel 1843), *Capoeta mandica* Bianco and Banarescu 1982, and *Capoeta barroisi* Lortet 1894. The taxonomic status of the recently described *Capoeta turani* Özulug & Freyhof, 2008; was not well supported by our data. Genetic distances among these subclades ranged from 1.1% to 1.7% (Table S4). Species delimitation methods recognized *C. mandica*, *C. barroisi*, *C. trutta*, and *C. turani* as valid, even given the relatively low genetic distances separating them.

### 3.2 | Aralo-Caspian clade

The Aralo-Caspian clade comprised populations of rivers that flow to the Aral, Orumieh, and Caspian seas, and several rivers in central Iran. This was a well-supported clade separated into three subclades (Fig. 4) with genetic distances among species ranging from 1.1% to 3.7% (Table S4).

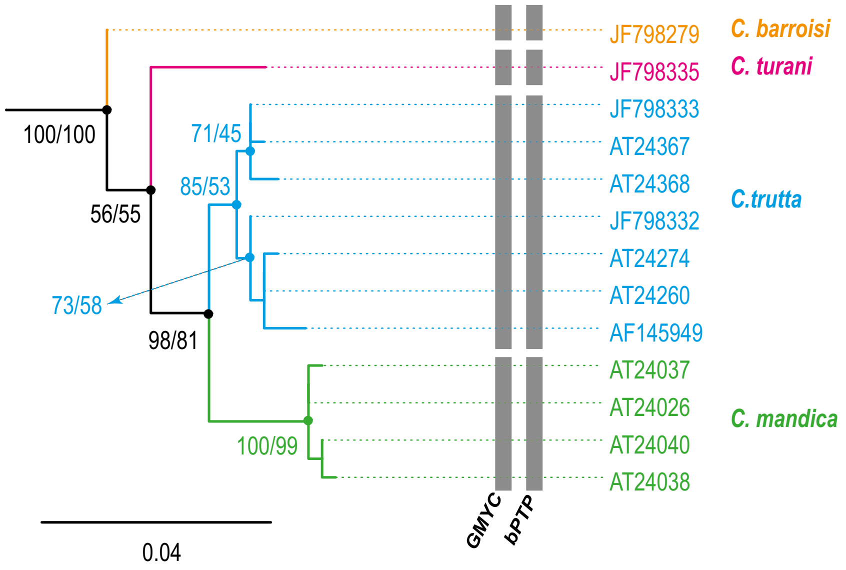

**FIGURE 3** Mesopotamian clade. Values at nodes correspond to BI posterior probability/ML bootstrap. The left bar indicates species delimited with General Mixed Yule-Coalescent and the right bar species delimited with bPTP. Sample codes correspond to GenBank Accession Numbers or Haplotype codes

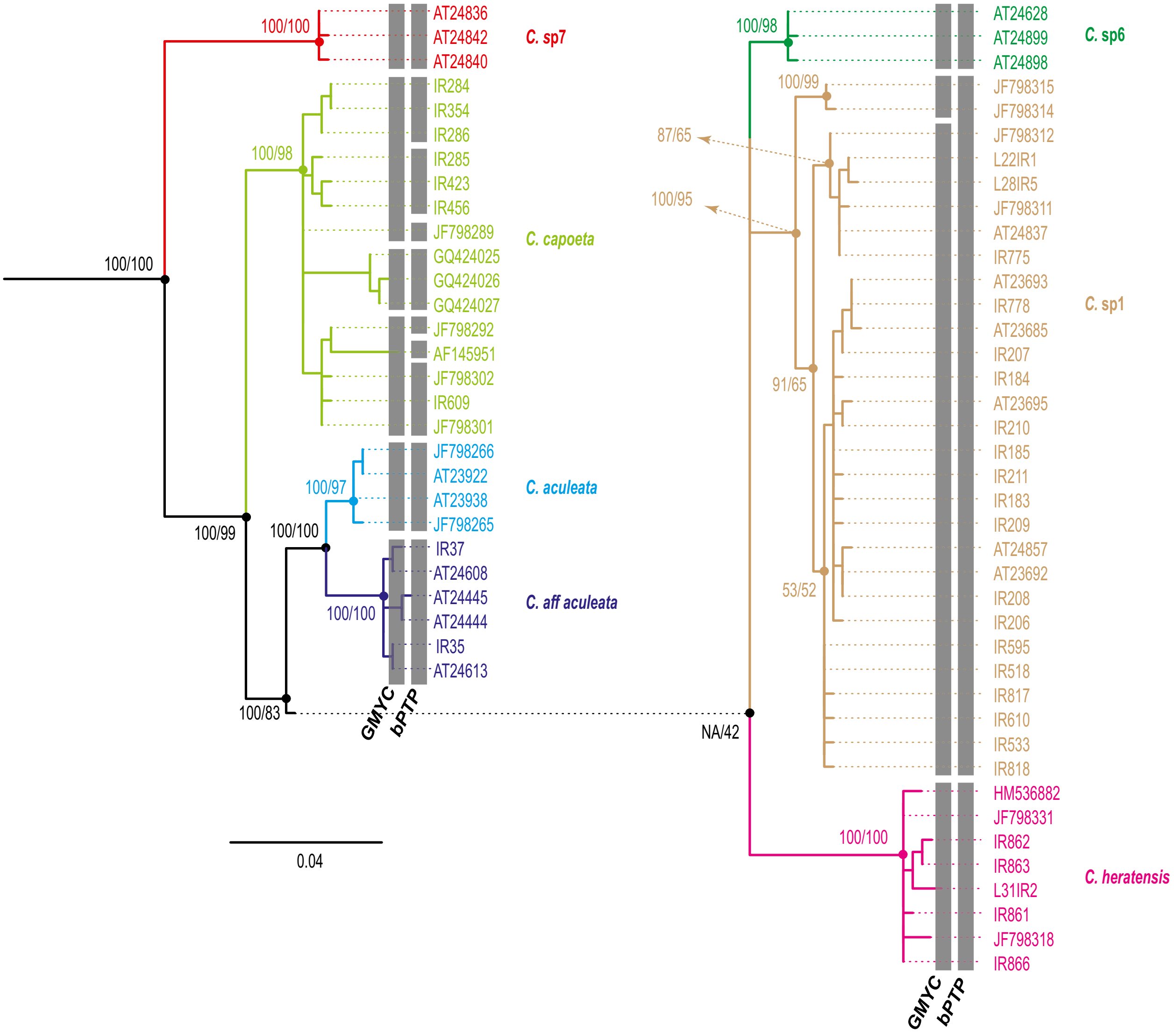

**FIGURE 4** Aralo-Caspian clade. Values at nodes correspond to BI posterior probability/ML bootstrap. The left bar indicates species delimited with General Mixed Yule-Coalescent and the right bar species delimited with bPTP. Sample codes correspond to GenBank Accession Numbers or Haplotype codes

The most basal subclade (*Capoeta* sp7) comprised specimens from the Tejan River (Caspian basin), which could not be attributed to any described species.

The second subclade included *Capoeta capoeta* (Güldenstädt 1773), *Capoeta sevangi* De Filippi, 1862, and *Capoeta ekmekciae* Turan, Kottelat, Kirankaya, and Engin, 2006, from Turkey, northwestern Iran, and Armenia (Fig. 5). Phylogenetic relationships within this subclade were unresolved, and genetic distances between species were low (0.7%). Results of the GMYC and bPTP differed for this subclade, suggesting the use of a genetic marker more sensitive to population differences. Sequences of *C. ekmekciae* were clustered together, and both species delimitation methods recognized it as a distinct evolutionary unit, which, along with the low genetic differences within this subclade (0.7%), could possibly be attributed to population differences within the same species. Interestingly, a specimen of this subclade was captured in the Caspian basin, which is geographically distant from the locations in which the other specimens were obtained, that is, chiefly the Orumieh basin and other areas in Turkey. This sample was checked twice to be sure that the result is not due to contamination (we repeated the DNA extraction, amplification and sequencing). The lack of more specimens presenting the same condition prevented us to reach any conclusion on it, so we prefer to interpret the data literally until we find more specimens and we study more in depth this question.

The third subclade included populations from north and northeastern river basins of Iran and river basins of Turkmenistan. We found two groups separated by genetic distances varying from 1.1% to 2.9%. The first consisted of two well-supported subgroups, *Capoeta aculeata* (Valenciennes 1844) occurring in the Karun and Kor basins, and a second occurring in the Karkheh River of the Tigris basin (*C. aff aculeata*). Both GMYC and bPTP identified the subgroups as distinct taxonomic units. The second group comprised three well-supported subgroups

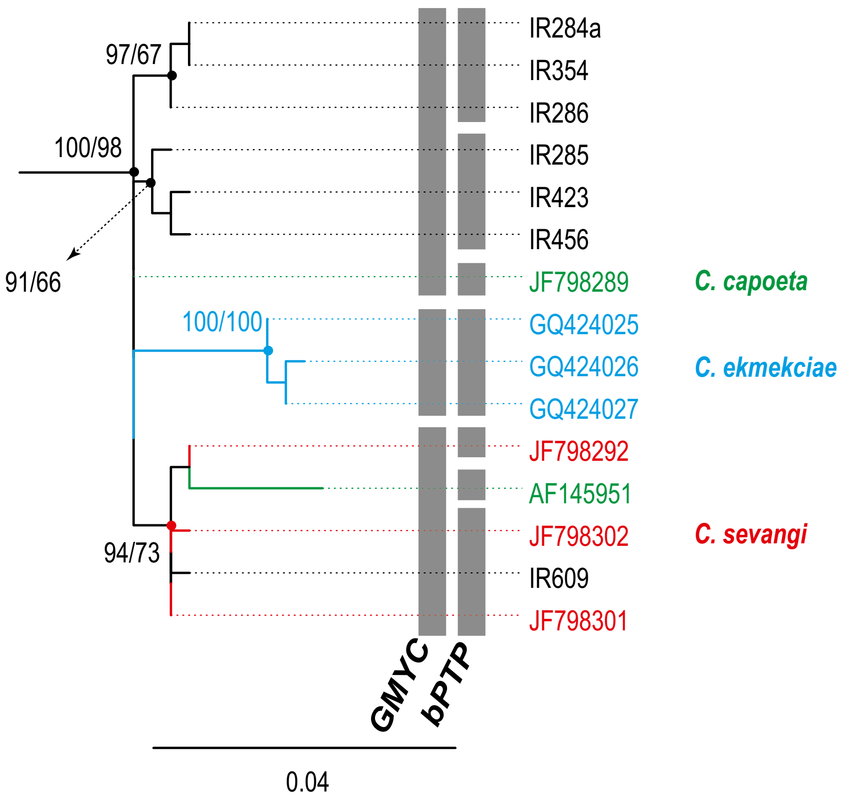

**FIGURE 5** *Capoeta capoeta* species complex. Values at nodes correspond to BI posterior probability/ML bootstrap. The left bar indicates species delimited with General Mixed Yule-Coalescent and the right bar species delimited with bPTP. Sample codes correspond to GenBank Accession Numbers or Haplotype codes occurring in the Caspian, Tedzhen, and Namak basins. Mean genetic distances between sequence pairs within this group ranged from 1.4% to 2.5%. Relationships within this group were unresolved. One subgroup was found in southeastern regions of the Caspian basin and in the Tedzhen basin, which is located mainly in Turkmenistan and Afghanistan. For this subgroup, *Capoeta heratensis* (Keyserling 1861) is suggested as the valid name, as the species was described from specimens caught in a river near Herat in Afghanistan. The other two subgroups, belonging to Iranian populations with southern Caspian distribution, could not to be assigned to any described species. One was recognized by Levin et al. (2012) and designated *Capoeta* sp1. We retain this nomenclature for populations of the southern Caspian basin. The third subgroup occurred in Namak endorheic basin from two geographically close but separated rivers, the Jajrud and Namrud Rivers, and is herein referred to as *Capoeta* sp6. Both GMYC and bPTP recognized *C.* sp6, *C.* sp1, and *C. heratensis* as distinct species.

The haplotype network of the populations from the northern and northeastern river basins demonstrates clear structuring

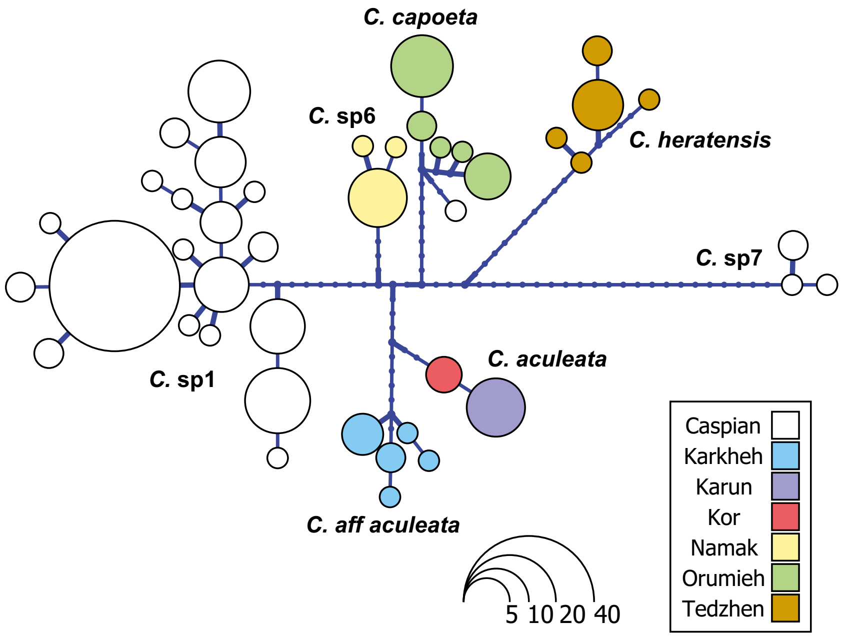

**FIGURE 6** Haplotype networks of available specimens of the Aralo-Caspian clade among basins (Fig. 6). No haplotypes were shared among populations of different basins. The group designated *Capoeta* sp1 was the most diverse taxon in this network, showing 19 haplotypes. *Capoeta aculeata* presented the lowest diversity, with two detected haplotypes. It is possible that the bigger number of samples for *C.* sp1 is biasing a little our results in some grade, but mostly, we believe that *C. aculeata* is living in a very arid region with very strong fluctuations on the water level (specially the population in Kor basin), so we suppose in their history they suffered many bottleneck events, which is not the case of *C.* sp1 which is present in a very humid region with high levels of precipitation. Also, we suppose that it have something to do with the fact that rivers in the Caspian basin, where *C.* sp1 is present, are all independent without freshwater connections what can help to keep rare alleles established in smaller independent habitats, which will show a higher diversity.

## 3.3 | Anatolian-Iranian clade

The Anatolian-Iranian clade, the sister clade of the Aralo-Caspian clade, includes species widespread throughout the Anatolian peninsula and river basins of western and central Iran. This well-supported clade was the most diverse among the *Capoeta*, comprising six subclades, with genetic distances ranging from 1.5% to 5.4% (Figs 2 and 7).

The first subclade consisted of *Capoeta sieboldii* (Steindachner 1864) from the Kelkit River in Turkey, which drains into the Black Sea.

A second subclade split into two well-supported groups separated by a genetic distance of 2.7%. The first included populations from Turkey and was attributed to *Capoeta bergamae* Karaman 1969, and the second, also from Turkey, was described by Levin et al. (2012) and designated *Capoeta* sp2. Both groups were recovered as valid species by both methods used.

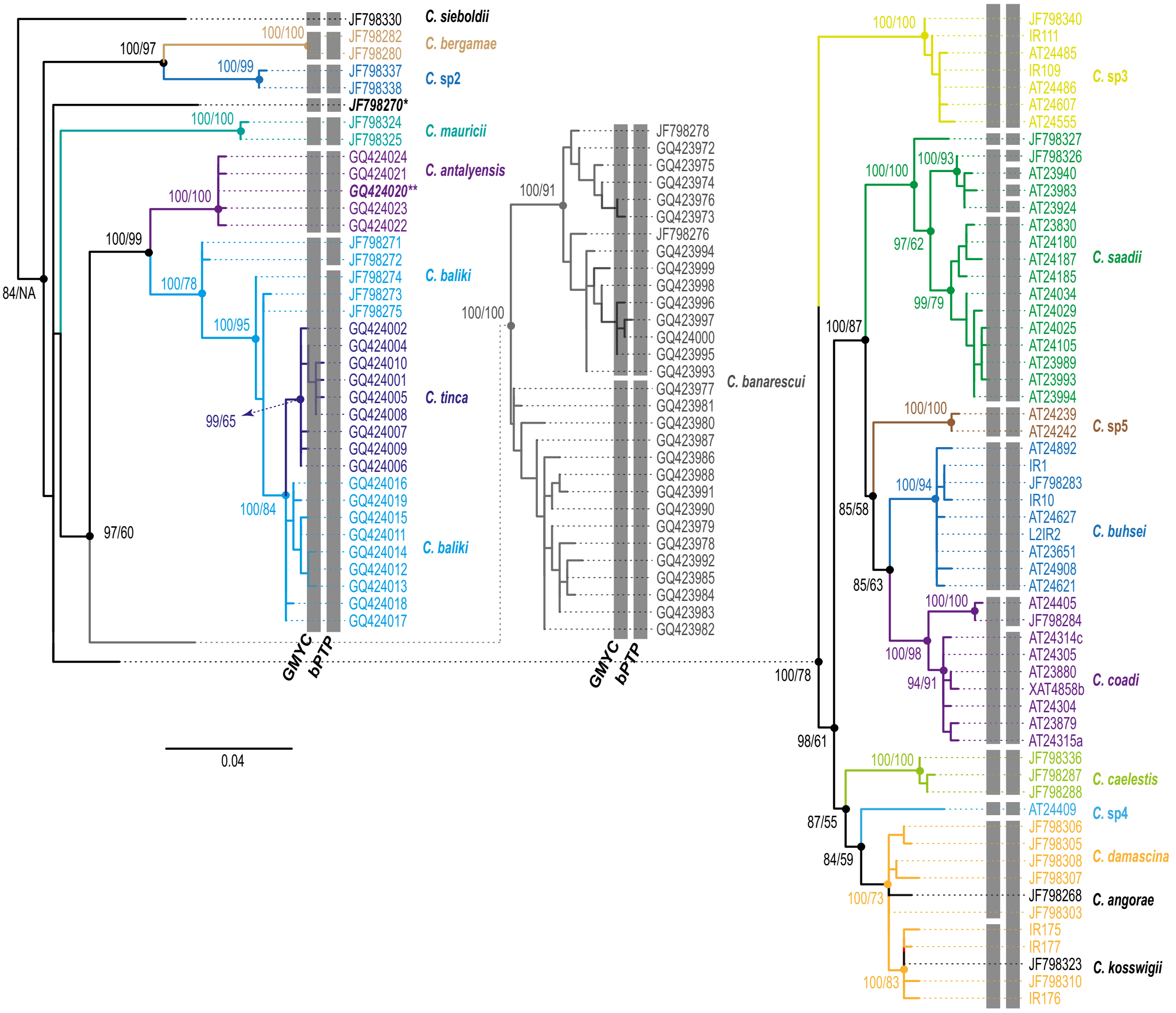

**FIGURE 7** Anatolian-Iranian clade. Values at nodes correspond to BI posterior probability/ML bootstrap. The left bar indicates species delimited with General Mixed Yule-Coalescent and the right bar species delimited with bPTP. Sample codes correspond to GenBank Accession Numbers or Haplotype codes

The third subclade included populations of *Capoeta mauricii* Küçük, Turan, Sahin, and Gülle 2009, inhabiting the Sarioz Stream and Eflatum Spring in the Beysehir Lake basin in southwestern Turkey.

The fourth subclade included *Capoeta antalyensis* (Battalgil 1943) from the Boga Cayi River in Turkey, near the type locality of the species. Again, both methods of species delimitation supported subclades three and four as valid species.

A fifth subclade was divided into two groups. The first group was formed by two well-supported subgroups. The first subgroup included samples identified as *C. antalyensis*. The genetic distance between sequence pairs within this subgroup was 0.2%, and both species delimitation methods considered the subgroup as a valid species. Hence, we tentatively interpret this as misidentification of these specimens, as samples of type locality of *C. antalyensis* were present in the fourth subclade. A second subgroup consisted of *Capoeta baliki* Turan, Kottelat, Ekmekçi, and Imamoglu 2006, and *Capoeta tinca* (Heckel 1843). However, the species delimitation methods used did not recover them as separate species. The second group of this subclade consisted of samples identified as *Capoeta banarescui* Turan, Kottelat, Ekmekçi, and Imamoglu 2006 and as *C. cf banarescui* by Levin et al. (2012). Both GMYC analysis and bPTP recognized two evolutionary units, one of which corresponded to *C. banarescui*.

The final subclade comprised two well-supported groups. One consisted of specimens from the Karkheh basin in western Iran. In a previous phylogenetic study, these fish were assigned to *Capoeta* sp3 (Levin et al., 2012). Both GMYC analysis and bPTP recognized it as a valid species. The second group of this subclade separated into two subgroups, the first found mainly in Turkey and the other primarily in Iran. The Turkish subgroup comprised two sets: *Capoeta caelestis* from the Ilica Stream in central Turkey and the Kargi Cayi River in southwestern Turkey, recognized as a single species by both species delimitation approaches, and a second set including *Capoeta damascina* (Valenciennes 1842); *Capoeta kosswigi*, Karaman 1969; *Capoeta angorae* (Hankó 1925); and two Iranian specimens from the Dez basin (Tigris tributary) not previously described (*Capoeta* sp4). Neither delimitation method recognized species differences, with the exception of *Capoeta* sp4, which both recognized as a valid species.

The second subgroup occurring mainly in Iran also split into two mitochondrial lineages. One included *Capoeta buhsei* Kessler 1877, of the Namak basin; *Capoeta coadi* Alwan, Zareian, and Esmaeili 2016, from the Tigris and Zayandeh Rud basins; and *Capoeta* sp5 not described previously, from the Zohreh basin. The methods used showed *C. buhsei* and *Capoeta* sp5 as valid species, but divided the *C. coadi* into two species, one occurring in the endorheic Zayandeh Rud basin and the other in the Karun basin of the Tigris drainage. However, the genetic distances were the lowest obtained in this analysis. The second mitochondrial lineage included *Capoeta saadii* (Heckel 1847), presenting strong geographic structuring. The GMYC method recognized three species: one in the Dalaki and Mand basins both draining into the Persian Gulf, one in the endorheic Kor basin, and one in the Rodan basin flowing into the Gulf of Oman. bPTP identified the four haplotypes of the Kor basin as separate evolutionary units, which we consider to be an artifact of the program. As the developers of both species delimitation methods recommend, their results have to

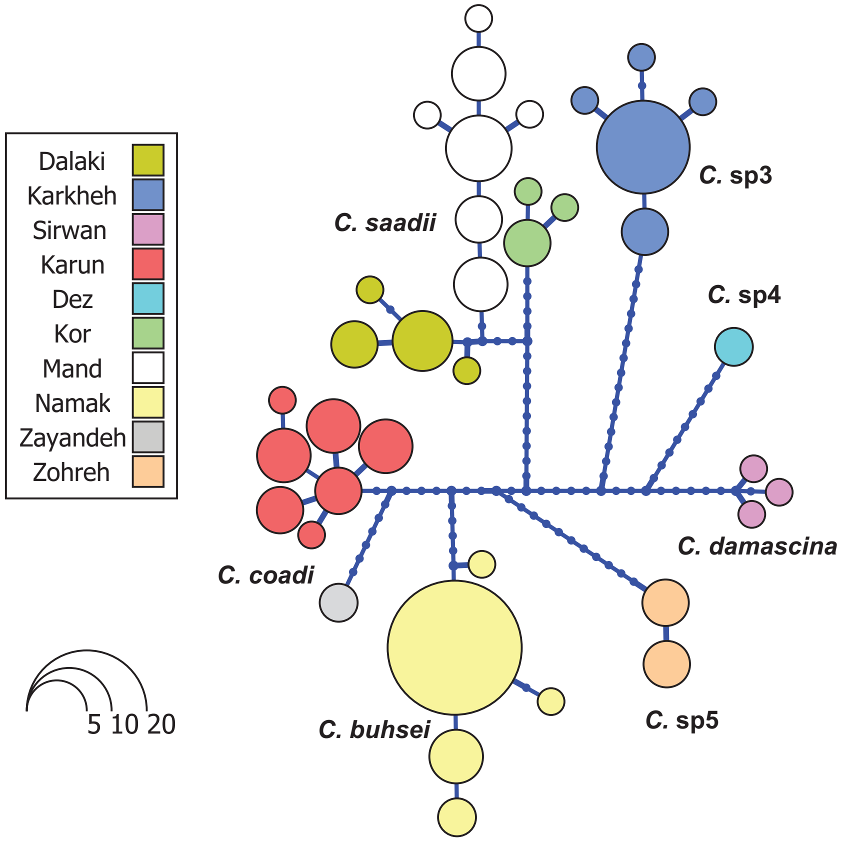

**FIGURE 8** Haplotype networks of available specimens of the Anatolian-Iranian clade be corroborated with other information sources. Here, in these four different "species" recognized by bPTP, all the information suggest that they are all the same species: GMYC does not recognize different species, genetic distances between them are very low, lower than some other within species distances in the genus, all samples come from the same sampling point, and they form all together a well-supported clade very close to all other samples from this species. So rather than different species, we interpret the results on this group as highly structured populations within the same species.

The network analyses showed high haplotype diversity and strong geographic structuring in *C. saadii* in comparison with the remaining haplogroups, with 14 haplotypes present in three basins and no haplotype shared among basins (Fig. 8). Generally, all *Capoeta* Species occurring in Karun and Mand basins presented high haplotype diversity, with seven haplotypes of *C. coadi* in Karun and seven haplotypes of *C. saadii* in Mand.

Separation of *Capoeta* from *Luciobarbus* was estimated to take place during the Middle Miocene, ca. 17.5 Mya (17–17.9 Mya) (Fig. 9). The divergence of the three main clades of *Capoeta*, the Mesopotamian, the Aralo-Caspian, and the Anatolian–Iranian, was estimated to have occurred in the Mio-Pleistocene (15.6–12.4 Mya), with the Mesopotamian clade diverging from the two other clades ca. 15.6 Mya (13.8–17.2 Mya) and separation of the Aralo-Caspian and Anatolian-Iranian ca. 12.4 Mya (10.5–14.4 Mya).

## 4 | DISCUSSION

This study provides the most comprehensive molecular phylogenetic framework of the *Capoeta* species in Iran to date. *Capoeta* was found to be monophyletic, consisting of three highly divergent lineages, as previously reported (Levin et al., 2012; Zareian, Esmaeili, Heidari, Khoshkholgh, & Mousavi-Sabet, 2016). Within Iran, these lineages are represented by the Mesopotamian clade along with the Aralo-Caspian clade and its sister group, the Anatolian-Iranian clade (Levin et al., 2012; Zareian, Esmaeili, Heidari, et al. 2016). We observed a complex phylogenetic pattern for *Capoeta*, with the presence of new mitochondrial lineages that, in some cases, indicated the need for rearrangement of the current systematics of *Capoeta* genus the region (Table 2). This supports the premise that the biodiversity of the area has been underestimated (Coad, 2006) and highlights Iran as critical for diversification studies, as it represents an important area of faunistic interchange among biogeographical regions (Kapli et al., 2015). Our molecular clock dates the separation between *Capoeta* and *Luciobarbus* at *ca.* 17.5 Mya, in the Middle Miocene. This estimate predates the divergence time of 13.9 Mya previously obtained for both genera. Based on the divergence time estimates, the separation of the main clades within *Capoeta* occurred during the Mio-Pliocene (15.6–12.4 Mya) period.

### 4.1 | Taxonomy and species relationships of *Capoeta* in Iran

The phylogenetic analyses and the GMYC/bPTP clustering methods agreed in most cases and suggested novel taxonomic findings within *Capoeta*. With multiple species-level taxa within its nominal species, *C. capoeta* forms a species complex. We propose a new taxonomic status for *C. mandica* and recognize new candidate species *C.* sp4, *C.* sp5, *C.* sp6, *C.* sp7, and *C. aff aculeata* (Table 2). The species *C.* sp1, *C.* sp2, and *C.* sp3 previously proposed by Levin et al. (2012) were supported by our analyses. Twenty-six *Capoeta* species, including the eight species (*C.* sp1, *C.* sp2, *C.* sp3, *C.* sp4, *C.* sp5, *C.* sp6, *C.* sp7, and

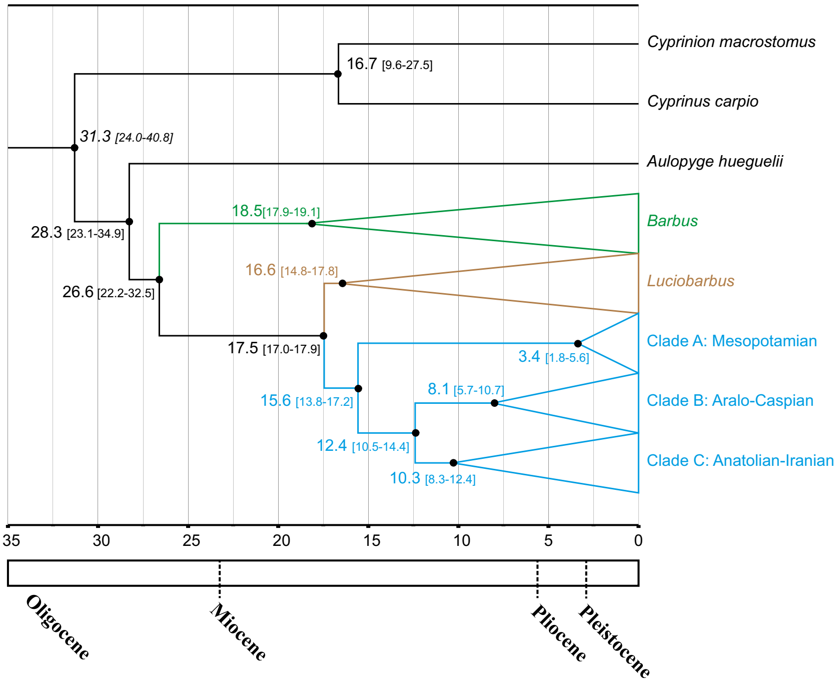

**FIGURE 9** Divergence time (Mya) and credibility intervals (95% highest posterior density)

**TABLE 2** Proposed systematic nomenclature for *Capoeta* species

| | Species of genus Capoeta that appear in the literature | Esmaeili et al. (2010) | Jouladeh-Roudbar et al. (2015) | Froese & Pauly (2016) | This study |
|---|---|---|---|---|---|
| Mesopotamian clade | *C. barroisi* | *C. barroisi* | – | *C. barroisi* | *C. barroisi* |
| | *C. mandica* | | *C. mandica* | | *C. mandica* |
| | *C. turani* | – | – | *C. turani* | *C. turani* |
| | *C. trutta* | *C. trutta* | *C. trutta* | *C. trutta* | *C. trutta* |
| | *C. anamisensis* | – | – | – | – |
| Aralo-Caspian clade | *C. ekmekciae* | – | – | *C. ekmekciae* | *C. capoeta* |
| | *C. sevangi* | – | – | *C. capoeta* | |
| | *C. capoeta* | *C. capoeta* | *C. capoeta* | | |
| | *C. heratensis* | | *C. heratensis* | | *C. heratensis* |
| | *C. Sp1** | | *C. gracilis* | | *C. sp1** |
| | *C. Sp6* | – | – | – | *C. sp6* |
| | *C. Sp7* | – | – | – | *C. sp7* |
| | *C. aculeata* | *C. aculeata* | *C. aculeata* | *C. aculeata* | *C. aculeata* |
| | *C. aff aculeata* | | | | *C. aff aculeata* |
| Anatolian-Iranian clade | *C. sieboldi* | – | – | *C. sieboldi* | *C. sieboldi* |
| | *C. bergamae* | – | – | *C. bergamae* | *C. bergamae* |
| | *C. Sp2** | – | – | – | *C. sp2** |
| | *C. mauricii* | – | – | *C. mauricii* | *C. mauricii* |
| | *C. antalyensis* | – | – | *C. antalyensis* | *C. antalyensis* |
| | *C. baliki* | – | – | *C. baliki* | *C. tinca* |
| | *C. tinca* | – | – | *C. tinca* | |
| | *C. banarescui* | – | – | *C. banarescui* | *C. banarescui* |
| | *C. Sp3** | – | – | – | *C. sp3** |
| | *C. Sp5* | – | – | – | *C. sp5* |
| | *C. buhsei* | *C. buhsei* | *C. buhsei* | *C. buhsei* | *C. buhsei* |
| | *C. coadi* | | | | *C. coadi* |
| | *C. caelestis* | – | – | *C. caelestis* | *C. caelestis* |
| | *C. Sp4* | – | – | – | *C. sp4* |
| | *C. saadii* | *C. damascina* | *C. saadii* | *C. damascina* | *C. saadii* |
| | *C. damascina* | | *C. damascina* | | *C. damascina* |
| | *C. kosswigi* | – | – | *C. kosswigi* | |
| | *C. angorae* | – | – | *C. angorae* | |
| N/A | *C. fusca* | *C. fusca* | *C. fusca* | *C. fusca* | – |
| | *C. pestai* | – | – | *C. pestai* | – |
| | *C. umbla* | – | – | *C. umbla* | – |
| | *C. erhani* | – | – | *C. erhani* | – |

In undescribed species with a mark (*), the name used by Levin et al. (2012) is kept in this study. N/A is stated for those species which have no information on the clade they belong to.

*C. aff* aculeata) proposed as candidate species, covering mostly of the genus distribution in Iran were recognized (Table 2).

### 4.1.1 | Mesopotamian clade

The taxonomic validity of *C. mandica* in the Mesopotamian clade has been questioned. Some authors consider it a subspecies of *C. barroisi* (Coad, 2015), whereas others consider it a different species (Esmaeili, Coad, Gholamifard, & Teimory, 2010; Jouladeh-Roudbar, Vatandoust, Eagderi, Jafari-Kenari, & Mousavi-Sabet, 2015; Özulug & Freyhof, 2008). Bayesian reconstruction provided evidence for the presence of a mitochondrial lineage distinct from *C. barroisi*. *Capoeta mandica* lineage appeared to be closely related to *C. trutta* and distantly related to *C. barroisi*, which led us to propose it as a valid species. The results of GMYC and bPTP were also congruent with the recognition of *C. mandica* as a separate species.

However, species of the Mesopotamian clade show low genetic distance and wide distribution. Hence, further investigation, including morphological characters and examination of more specimens throughout its population distribution, is needed to establish a robust taxonomy for this clade.

#### 4.1.2 | Aralo-Caspian clade

Within the Aralo-Caspian clade, a population from the Tejan River in the Caspian slope, sampled for the first time, was highly divergent from the remaining lineages. As the haplotype network analyses and the GMYC and bPTP methods for species delimitation also supported the differentiation of this lineage, we putatively considered it here as new species (*Capoeta* sp7). However, given that the *C.* sp7 lineage occurs in sympatry with *C.* sp1, further morphological analyses and additional samples from the region should be included to determine the origin of speciation, possibly introgressive hybridization, as reported in other cyprinids (Durand, Unlü, Doadrio, Pipoyan, & Templeton, 2000; Machordom, Berrebi, & Doadrio, 1990).

The populations identified as *C. capoeta* from western Caspian were not monophyletic and showed a complex phylogeny including two previously described species, *C. sevangi* and *C. ekmekciae*. This, together with the low phylogenetic resolution of this subclade, suggest these three species as a *C. capoeta* complex, calling for further study.

*Capoeta aculeata* showed two well-supported groups within southwestern basins of Iran, one in Karun and endorheic Kor basins and a second in the Karkheh basin. The Kor endorheic basin drains into Bakhtegan Lake, while Karun and Karkheh belong to the main Tigris basin and drain into the Persian Gulf. Even with relatively high haplotypic diversity, no shared haplotypes were found between basins, showing genetic separation of these populations and suggesting further study of species status of populations designated *Capoeta aff. aculeata*. Results of species delimitation methods also supported consideration of the populations from Karkheh basin as a different species. This points to an unique dynamic in large rivers such as those of the Tigris basin, in which independent regions are defined based on the sub-basins that can represent barriers to genetic flow among populations.

The existence of two species of *Capoeta* in the endorheic basin of Namak Lake in central Iran was supported by the phylogeny and species delimitation analyses. *Capoeta buhsei* included in the Anatolian-Iranian clade was previously recorded in this basin, with the type locality in the Karaj River near Tehran, Kessler 1877. The second, belonging to the Aralo-Caspian clade, was identified here for the first time and designated *Capoeta* sp6.

Three species of the genus *Capoeta* were found In the Caspian basin: *C. capoeta*, *C.* sp1 previously reported by Levin et al. (2012), and the newly identified *C.* sp7. The Caspian basin has a long history of fluctuation in sea level caused by climatic changes during Plio-Pleistocene (Mamedov, 1997). This complex history probably is one of the causes of the structure observed in our phylogeographic analyses of the populations of *C.* sp1 belonging to Aralo-Caspian clade, which likely represents connection and isolation of rivers when the levels of the Caspian Sea changed during the last glaciations.

#### 4.1.3 | Anatolian-Iranian clade

The Anatolian-Iranian clade was found to be the most widespread and diversified clade of *Capoeta*, as was previously reported (Levin et al., 2012). This was a similar pattern to that reported in other genera from Anatolia and western Iran, such as *Mesalina* (Kapli et al., 2015), *Mauremys* (Vamberger et al., 2013), *and Trachylepis* (Fattahi et al., 2014). These wide distributions are probably due to recent dispersion events and/or lower barriers for some species groups.

Although *C. damascina* shows a broad distribution in Turkey, we found only a few specimens of this species in the Sirwan basin (Tigris tributary) in Iran. Some populations previously described as *C. angorae* and *C. kosswigi* are here considered synonymous with *C. damascina*, as our species delimitation methods did not indicate differences.

Nevertheless, our analysis separated populations of *C. buhsei* and recently described *C. coadi* (Alwan, Zareian, & Esmaeili, 2016) into two groups: the first clustered populations from the endorheic Namak basin and a second clustering those from the Karun and Zayandeh Rud basins. Our species delimitation methods suggested two species with interruption of genetic flow during the middle Pliocene. This temporal isolation during the middle Pliocene of the Namak basin is also reflected in species *C.* sp6 of the Aralo-Caspian clade. While the fauna in the Namak basin may have been affected by recent influences (Berg, 1940), Pliocene origin of the freshwater fish fauna in Namak basin has also been suggested for *Salmo trutta* (Derzhavin, 1934). Early Miocene deposits of Foraminifera indicate a hypersaline lagoon or inner shelf marine environment and humid climate during the Pliocene possibly changed the hydric balance and the salinity of Namak Lake (Daneshian & Dana, 2007; Zhu et al., 2007).

Populations of *Capoeta* from the Zohreh basin in the slope of the Persian Gulf (Fig. 8) belonged to *C.* sp5. The isolation of this population could be related to the formation of fluvial basins in Iran during the Pliocene. Populations of *C. saadii*, inhabiting areas south of the range of *C.* sp5, present well-distinguished structure also corresponding to the Pliocene period, during which the populations of the Mand and Dalaki basins flowing to Persian Gulf became isolated from the endorheic Kor basin (Hrbek & Meyer, 2003; Teimori, Esmaeili, Gholami, Zarei, & Reichenbacher, 2012).

### 4.2 | Hypothesis for the origin of *Capoeta* in Iran

We found clear correspondence between geographical and genetic origin of the clades, seeming to follow a south–north pattern of distribution. The most highly diverged Mesopotamian clade occupied the southern regions with the Aralo-Caspian clade in the northern regions and the Anatolian-Iranian clade in the middle regions being the most widespread. The Anatolian-Iranian clade overlapped with the other clades at the borders of their distribution. This is probably due to recent dispersion events of the Anatolian-Iranian clade.

The divergence of the three main *Capoeta* lineages was estimated to have occurred around 15.6–12.4 Mya. The uplift of the Zagros Mountains *ca.* 35–20 Mya in southern Iran and their stabilization ca.12.4–10 Mya (Mouthereau, 2011) and the uplift of the Alborz Mountains *ca.* 20–17 Mya (Ballato et al., 2008, 2010) in northern Iran correlate with the divergence times of the three main *Capoeta* clades. These major mountainous systems likely acted as barriers to dispersion of the fauna and flora of the region (Ansell et al., 2011; Feldman & Parham, 2004; Hrbek & Meyer, 2003; Kapli et al., 2015; Parsa, Oraie, Khosravani, & Rastegar-Pouyani, 2009; Rastegar-Pouyani, Rastegar-Pouyani, Kazemi Noureini, Joger, & Wink, 2010; Šmíd & Frynta, 2012; Vamberger et al., 2013). Secondary dispersions, especially noticeable in the Anatolian-Iranian lineage and less obvious in Aralo-Caspian lineage, shaped the current picture of the distribution of the clades.

To decipher evolutionary and biogeographical patterns in *Capoeta*, previous authors have calibrated a molecular clock for the cytochrome *b* gene using fossil records (Levin et al., 2012). According to the molecular evolutionary rate based on a relaxed molecular clock, *Capoeta* arose in the middle Miocene 17.5 Mya when the Gomphotherium Landbridge was an important route of terrestrial fauna exchange between Africa and Asia (Harzhauser et al., 2007; Rögl, 1999). This period of middle-to-late Miocene was marked by the alternating periods of closure of the Tethys Sea and probably explains the split of the three main *Capoeta* clades, which is concurrent with the early development of Zagros Mountains, influencing the separation of basins and populations, especially in Iran. The Zagros Mountain uplift began in the mid-Miocene as a result of tectonic activity primarily resulting from contact of the Iranian and Arabian plates (Molnar, 2006). During the formation of Zagros Mountains, new freshwater bodies, along with new barriers within existing basins, shaped the generation of the main lineages, as supported by our results. This suggests major tectonic processes as the main speciation force within *Capoeta*, which may also be applicable to other freshwater groups.

## 4.3 | Conservation

As mentioned before, there is an urgent need to assess the conservation status of these species and freshwater fauna in general in Iran. In general, main threats affecting *Capoeta* genus seem to be the water abstraction for irrigation projects and other human needs and habitat loss, especially in a mainly arid region which become dryer and warmer every year with less precipitations. In addition, this already fragile environment is also affected by the industrialization and constructions and pollutions related to it, which will have certainly a major role as a threat for all freshwater fauna in a developing country and show the need of more conservational controls and policies. Finally, there is also a very important impact on the local fauna caused by the invasive species, mainly commercially interesting species for human use as food. More regulations and a better environmental management are necessary in the country to preserve the rich and unique fauna living in the region.

## ACKNOWLEDGMENTS

We are grateful to Diushi Keri Corona Santiago for collection support and data analysis. This research was funded by the project of the Spanish Ministry of Economy and Competitiveness (CGL2013-41375-P) to Dr. Ignacio Doadrio.

## CONFLICT OF INTEREST

None declared.

## FUNDING INFORMATION

Spanish Ministry of Economy and Competitiveness, (Grant/Award Number: “CGL2013-41375-P”).

## SUPPORTING INFORMATION

Additional supporting information may be found in the online version of this article:

**How to cite this article:** Ghanavi, H. R., Gonzalez, E. G., and Doadrio, I. (2016), Phylogenetic relationships of freshwater fishes of the genus *Capoeta* (Actinopterygii, Cyprinidae) in Iran. Ecology and Evolution, 6: 8205–8222. doi: 10.1002/ece3.2411
